# What motivates medical students to learn about traditional medicine? A qualitative study of cultural safety in Colombia

**DOI:** 10.5116/ijme.5eb4.620f

**Published:** 2020-06-22

**Authors:** Juan Pimentel, Iván Sarmiento, Germán Zuluaga, Neil Andersson

**Affiliations:** 1 CIET-PRAM, Department of Family Medicine, McGill University, Quebec, Canada; 2 School of Medicine and Health Sciences, Del Rosario University, Bogotá, Colombia; 3 Centre for Tropical Disease Research (CIET), Autonomous University of Guerrero, Mexico

**Keywords:** Transformative learning, traditional medicine, participatory research, cultural safety

## Abstract

**Objectives:**

This study explored motivation dynamics of medical students engaging with traditional medicine in Colombia.

**Methods:**

We conducted a qualitative descriptive study as part of a larger participatory research effort to develop a medical education curriculum on cultural safety. Four final-year medical students participated in a five-month program to strengthen knowledge of traditional medicinal plants with schoolchildren in Cota, a municipality outside Bogota with a high proportion of traditional medicine users. Students and schoolteachers co-designed the program aimed to promote the involvement of school children with traditional medicine in their community. The medical students shared written narratives describing what facilitated their work and discussed experiences in a group session. Inductive thematic analysis of the narratives and discussion derived categories of motivation to learn about traditional medicine.

**Results:**

Five key learning dynamics emerged from the analysis: (1) learning from/with communities as opposed to training them; (2) ownership of medical education as a result of co-designing the exercise; (3) rigorous academic contents of the program; (4) lack of cultural safety training in university; and (5) previous contacts with traditional knowledge.

**Conclusions:**

We identified potential principles for engaged cultural safety training for medical students. We will use these in our larger training program. Our results may be relevant to other researchers and medical educators wanting to improve the interaction of medical health professionals in multicultural settings with people and communities who use traditional medicine. We expect these professionals will be better prepared to recognize and address intercultural challenges in their clinical practice.

## Introduction

Since 1977,^1^ the World Health Organization has called for engagement between Western and traditional medicine.^2^^,^^3^ The Alma Ata view of primary health care recognized that traditional practitioners and medicinal plants might have a role in health promotion.^4^^,^^5^ Culturally diverse Colombia is a suitable setting for medical education research, with potential lessons for other countries. The Colombian government supports official health services based exclusively on the Western biomedical model, yet a high proportion of the population uses traditional medicine.^2^ The cultural gap between health professionals and communities, who include different cultures, hinders community access to acceptable and effective health services.^6^

Along with many other countries,^7^ Colombian legislation ratifies a commitment to primary health care and to include the intercultural approach as a core element of health development (Article 13, law 1438 of 2011). This legislation has not permeated everyday medical practice, however, and medical education still lacks training programs to address cultural differences between patients and practitioners. Uninformed intercultural interactions hinder the delivery of culturally safe health services.^8^^,^^9^

As a partial response to this state of affairs, there have been several global calls for awareness in medical education of the cultural preferences of local communities.^10^^,^^11^ Cultural competence is currently a common intercultural approach in medical education.^12^ Its utilitarian understanding of culture and centering on the dominant culture, however, has led educators to advocate for more comprehensive approaches to health care, such as cultural safety.^13^

Cultural safety training promotes professional practice “that is spiritually, socially, emotionally and physically safe for people; where there is no assault, challenge or denial of their identity, of who they are, and what they need.”^14^ Recent evidence links this type of education to improved relationships between health professionals and their culturally different patients, with improved health outcomes.^14^

Inspired by the cultural safety approach, we are developing a medical curriculum in Colombia to promote respect for traditional medicine users.^15^ The important challenges, however, are that health professionals have no motivation to interact with traditional medicine,^16^ and they receive no cultural safety training. This small exploratory study looks for motivating dynamics that might inform the larger participatory development of a cultural safety curriculum, increasing medical student interaction with traditional medicine. By working with and learning from traditional medicine users, we expected the medical students to become more respectful of and more open to traditional medicine practices and users.

## Methods

### Study design and participants

We conducted a qualitative descriptive study. Four final-year medical students (two women and two men, age range 22-24 years) attending a course on community health^17^ and two instructors conducted a community-based learning exercise in a public school and a private preschool in the municipality of Cota, Colombia between July and November 2015. The details of the community health course are described elsewhere.^17^

This community exercise embraced the bioethical principles proposed by the Council for International Organizations of Medical Sciences,^18^ the Declaration of Helsinki,^19^ the resolution 8430 of 1993 of the Republic of Colombia, and the principles for studies with indigenous communities in that country.^20^ We made clear to participating medical students that the exercise was not to provide health services to the schools nor to train the medical students in the use of traditional medicine.

Medical students provided written informed consent. We obtained parental consent during a meeting at the beginning of the school year. In the meeting, two researchers presented the project to the parents and explained that it was co-designed and supervised by the teachers as part of the official curriculum. The Sub-committee for Research of the Faculty of Medicine at La Sabana University provided ethical approval of the project (approval number: 445).

### Setting

Cota, a small municipality 15km from the capital of Colombia, includes a high proportion of traditional medicine users, many of them subsistence farmers who identify with both indigenous and European health traditions. A local committee, including traditional medicine users who were also schoolteachers, and a local NGO (Center for Intercultural Medical Studies – CEMI), supported the exercise.

Prior to the fieldwork, the medical students received training using standard medical school lecture formats on elements of cultural safety, traditional medicine, primary health care, and participatory research. A series of planning meetings increased the trust between the medical students and the Cota community. In parallel, to increase their knowledge of the educational content, the students conducted a literature review on interventions aimed at strengthening traditional knowledge, using PubMed, LILACS, and Google Scholar. Finally, the community and teachers at the local schools discussed the perceived needs of the community regarding the recovery of traditional medicine knowledge and resources.

School teachers helped the medical students develop a list of opportunities for accessing lost traditional knowledge among the children attending the schools. The medical students and school teachers prioritized the opportunities using the Hanlon method^21^ and used a problems and objectives tree to analyze the selected issue and to propose solutions.^22^ Finally, the students and teachers created a work plan that followed the logical framework approach,^23^ and scheduled a time commitment for four hours per week over four months. The schoolteachers, medical students, and instructors developed 33 activities aligned with the ages of the children who participated in the project (Table 1).

The project promoted the intergenerational transmission of knowledge between groups of children and their parents through surveys with open-ended questions that the learners asked their parents at home. The intended effect was that, by asking the questions, children would recognize there was an issue and discuss it with their families, peers, and community. With the permission of school principals, the medical students also helped the children to restore school gardens of medicinal plants. The school gardens offered an opportunity for children to take care of the plants used for in-class discussions, thus promoting hands-on experience.

The medical students conducted the 33 planned activities (for example, recognition and care of medicinal plants, and visiting the Cota’s medicinal garden), accumulating 64 hours of direct work with the community. Some 482 people benefited from the project either directly or indirectly. The characteristics of the beneficiaries are shown in Table 2.

At the end of the exercise, the medical students organized an activity they called Plant-art to share the results with the school community; the participating children made infographics to communicate their experience and the results of their research (Table 3).

**Table 1:** Number of activities and their specific objective for each group of learners, Cota (Colombia), 2015

Grade	Number of activities	Objective	Themes
Ninth	Five^*^	Improve the application of concepts and practices of traditional self-care for their daily lives	Women's health care, self-care for the common cold, self-care for diarrhoea, nutrition: basic concepts^†^
Seventh	Eleven^*^	Deepen the knowledge about medicinal plants and to introduce the concept of traditional self-care of health	Traditional knowledge, restoration of the orchard of medicinal plants, theatrical representation about self-care, caring for medicinal plants, visiting the medicinal botanical garden of Cota^†^
Sixth	Six^*^	Raise the interest of learners in medicinal plants and traditional knowledge	Sensorineural recognition of medicinal plants, restoration of the orchard of medicinal plants, caring for medicinal plants^†^
Transition	Eleven^*^	Raise the interest of learners in medicinal plants	Sensorineural recognition of medicinal plants, making and designing flowerpots, caring for medicinal plants, making seals with medicinal plants, visiting the medicinal botanical garden of Cota

*The designers set the number of activities before the exercise †The teachers and students defined the themes together

### Data collection

The medical students each wrote a brief narrative describing what facilitated their interaction with traditional medicine. They shared these texts with one another and participated in a group discussion of their reports, facilitated by educators from La Sabana University and CEMI.

**Table 2:** The beneficiary population of the community exercise, Cota (Colombia), 2015 (n=482)

Grade	Age range (years)	Males	Females	Direct Beneficiaries*	Indirect Beneficiaries†	Total
Transition	4 to 5	7	6	13	44	57
Sixth	12 to 13	17	15	32	122	154
Seventh	13 to 14	14	17	31	122	153
Ninth	15 to 21	15	16	31	87	118
Total	4 to 21	53	54	107	375	482

* Students of the school and preschool in Cota † People in the close family of the students in Cota

### Data analysis

We transcribed written information and audio files of the group discussion and submitted them to an inductive thematic analysis following the methods proposed by Braun and Clarke.^24^ Two researchers (JP and IS) independently reviewed the transcripts and defined codes, involving a third party (GZ) in instances of disagreement. Consequently, they compared their independent analysis and created themes and subthemes to offer a comprehensive explanation that would answer the research question.

**Table 3:** Selected themes submitted for the infographics presented in Plant-art, Cota (Colombia), 2015

Grade	Infographic theme
Ninth	Nutrition
	Women's care
	Self-care for the common cold
	Self-care for diarrhoea
Seventh	Manzanilla [Chamomile] (Matricaria chamomilla)
	Diente de león [Dandelion] (Taraxacum officinale)
	Sábila [Aloe vera] (Aloe vera)
	Cidrón [Lemon verbena] (Aloysia citriodora)
	Limonaria [Lemon grass] (Cymbopogon citratus)
	Caléndula [Marigold] (Calendula officinalis)
	Menta [Mint] (Mentha sp.)
	Canelón [Radiator Plant] (Peperomia inaequalifolia)
Sixth	Diente de león [Dandelion] (Taraxacum officinale)
	Manzanilla [Chamomile] (Matricaria chamomilla)
	Ortiga [Stinging nettle] (Urtica dioica)
	Limonaria [Lemon grass] (Cymbopogon citratus)

## Results

The medical students described what motivated them to explore traditional medicine. They appreciated learning from or with people, as opposed to training them. The shared ownership of medical education motivated them as students co-designed the exercise with the community. They preferred a rigorous structure of the academic content of the program. They noted a lack of cultural safety training programs at university. And the exercise helped them recognize their previous experience with traditional knowledge.

### Learning with the community

The students acknowledged gaps in their medical education as they became aware of the widespread traditional knowledge in the communities where they live and work:

“One feels a little bit uncomfortable because, this close to finishing our career, one realizes that there is some knowledge about health that we do not have.” [male, 23 years, No. 1]

It was interesting for students that, in traditional medicine, the production of knowledge occurs outside of the academic context of Western universities:

“We realized that the source of knowledge was not us but the community.” [female, 22 years, No.3]

“The surprise was that the majority of these children already knew these traditional concepts thanks to the intergenerational transmission of information in each family.” [male, 24 years, No.2]

They expressed surprise that the direction of knowledge transfer, which they originally expected to be from the university to the community. As the project advanced, information began to move in the opposite direction:

“That moment was uncomfortable and disconcerting: we realized we came to the community to develop this project with very little knowledge about traditional medicine. However, the children taught us many things we did not know.” [male, 24 years, No.2]

“Most of the traditional knowledge was imparted by the community […] an atypical flow of knowledge, from which we learned.” [male, 23 years, No.1]

This elicited student reflection on conventional research and health interventions in Western universities. The usual vertical approach to research was replaced by a more horizontal dialogue between the community and university students:

“They learned as much as we learned. That is not typical in research, where the researcher usually has way more knowledge and is, therefore, better able to intervene and teach. We experienced research-based on participation and dialogue, with the community and ourselves at the same level, both contributing knowledge to solve problems.” [female, 22 years, No.3]

“We found other sources of knowledge. The hands-on experience became very important. It was like learning a different language.” [female, 23 years, No.4]

The medical students gradually realized that their role as researchers in the project was mostly about respecting and thus strengthening knowledge and practices that had existed in the community for a long time:

“What we ended up doing was renovating interest in the knowledge they already had. It was like bringing that knowledge back to this century. We came here not to generate knowledge but to revitalize and to strengthen local knowledge, to foster the intergenerational flow of knowledge, and to encourage them to recover and promote their knowledge.” [male, 24 years, No.2]

“For the teachers and schoolchildren, the involvement of medical students boosted the process; they felt supported and encouraged to recover their traditions.” [female, 23 years, No.4]

### Ownership of medical education

Throughout their participation in the community-based experience, the students became more aware of the disconnect between medical education and the cultural context they work in:

“It is not that these communities live far from Western medicine, from the ‘real medicine.’ But many of us, health professionals and some citizens, live far from traditional medicine.” [female, 23 years, No.4]

“There are a tradition and history behind this topic, and the people have faith in that. One should be there to strengthen and not to change their culture.” [male, 23 years, No.1]

The medical students also reflected on the gap between the conventional care available in official health services and culturally safe care that health professionals could offer based on local resources and traditional knowledge.

“This training helps to reevaluate the field of health promotion because, at the hospital, health services are oriented towards disease. In this project, however, we learned there are many things that we can do in the community to promote health using local resources and traditional knowledge.” [male, 24 years, No.2]

The medical students mentioned the contributions of this community-based learning to their professional training. They saw traditional medicine as a key component of culturally safe health services in multicultural settings.

“I can now make recommendations [to patients] about self-care using traditional knowledge. It would be irresponsible to prescribe plants, but we can give some simple advice for health promotion based on traditional knowledge.” [female, 23 years, No.4]

A key experience was co-creation and co-ownership of elements of the training. By the end of the project, medical students began to implement some traditional care practices that they learned from the community:

“In nutrition, for example, traditional advice is to eat as a family. And when one does so, one realizes that there is much more behind eating as a family, such as strengthening ties. As a medical student, one eats very badly and following traditional advice could help to improve our nutrition. In the case of women's health, one can now give traditional advice to improve the health of women in our families.” [male, 23 years, No.1]

“It made me reflect on my own self-care. I realized that I needed to eat healthier, to do exercise, and to improve my relationships with my family and friends. Moreover, when I got a common cold, I did some traditional care, and I felt better, it was less severe.” [female, 22 years, No.3]

Finally, the students identified risks associated with a reckless approach to traditional medicine. For example, the distortion of traditional knowledge that frequently occurs by unscrupulous persons:

“We are aware that in this world of traditional knowledge, we must be very careful of charlatans. For example, in the case of neo-shamanism.” [male, 24 years, No.2]

“I believe this training is about health promotion because learning to manage medicinal plants involves much more complex knowledge and training.” [male, 24 years, No.2]

### Rigorous program structure

At the beginning of the community-based experience, the students reported an attitude of skepticism or caution regarding traditional medicine:

“I have never believed in medicine that is not Western medicine. Perhaps this is because all my life I have followed a line of thought that I see as strictly logical and scientific, without ever opening myself to knowledge fields that are not based on the Western scientific method.” [female, 23 years, No.4]

The students appreciated the lecture-based introduction because it gave them a recognizable format for organizing this new type of knowledge:

“I remember the first days of the training in which the professors gave us structured lectures on traditional medicine, participatory research, and intercultural dialogue. We realized that there are serious researchers from prestigious institutions who study and promote a respectful and rigorous approach to traditional knowledge.” [male, 24 years, No.2]

### Paucity of cultural safety training

Asked about the reasons for wanting to participate in the course, the students mentioned the lack of these experiences at the university level and their curiosity about exploring the ways traditional medicine users and their communities understand and approach health and disease:

 “Most physicians do not have the chance to explore local understanding of the health-disease phenomenon during training. These are different from those of the Western biomedical model.” [female, 22 years, No.3]

The content offered in the course proved to be novel for the students:

“It was something totally new for us.” [male, 23 years, No.1]

“At the beginning, traditional knowledge was a totally unknown type of knowledge for me.” [female, 23 years, No.4]

### Prior traditional medicine experience

Some students recognized their previous contacts with traditional knowledge, either through their family or close friends:

“I have an olfactory memory, and I recognized the plant that my grandmother used to give me when I was little, and I was sick.” [female, 23 years, No.4]

“Once when I was at my parent’s farm in Caquetá [Colombian region], my brother got diarrhea, and a local lady who used to work in the farm gave him a traditional remedy, and it helped.” [female, 22 years, No.2]

## Discussion

This study is part of a larger participatory research effort to develop a medical education curriculum on cultural safety.^15^ The idea is to encourage medical students to acknowledge and to respect patient ways of being and knowing. Training in cultural safety^25^ can bridge the divide between traditional medicine users and health professionals in Colombian health services. This in turn can improve multicultural patient access to health services and increase the overall quality of healthcare delivery.^14^ As a first step along this path, we explored what motivates medical students as they interact with traditional medicine. We identified several actionable themes: learning with the community, ownership of medical education, a rigorous structure of the training program, the paucity of cultural safety programs, and previous exposure to traditional medicine.

The community base of this training encouraged medical students to acknowledge traditional medicine users as a source of knowledge. Learning with the community promoted equitable inclusion of traditional medicine users. The participatory nature of the project contrasted in a positive way, the conventional hierarchy of knowledge passed from teacher to student.

At the beginning of the project, students expressed caution or even skepticism about traditional medicine since it does not follow familiar logic and or what they recognize as scientific procedures. Parra and Pacheco suggested this attitude stems from the proposal of conventional medical training that Western science is the only valid way to create and transfer knowledge.^26^

In our study, the focus on community voices provoked a positive attitude of the medical students towards traditional medicine. Macaulay^27^ highlights the value of community-based participatory initiatives that allow researchers and communities to contribute equally to co-creation of knowledge. Researchers and communities learn from each other. Participatory research combines education and action to “democratize the knowledge production process.”^28^

Several authors have described the role of community-based projects in intercultural training. Clark^29^ reported that social work students increased their personal and professional awareness of diversity, recognized their own biases, and increased their level of comfort with cultural differences after participating in community-based transformative learning. Muñoz-Cano^30^ and Arce-Antezana^31^ suggested that project-based learning allows the development of the skills for intercultural education by promoting awareness and respect for other cultures, languages, and people; it allows medical students to empathize with people who are different from themselves. Our study confirms the value of community-based learning in cultural safety.

Engaging higher education students in the co-creation and co-ownership of academic content has recently gained attention,^32^ although it is still uncommon in medical education. Curriculum co-creation is additionally promoted by the World Federation of Medical Education.^33^

Benefits from co-created curriculum initiatives include a deeper understanding of the learning process, changing the way students relate to others, and enhanced student motivation, enjoyment, and enthusiasm for learning.^34^

By co-designing the exercise with community members and schoolteachers, the students in our study explored traditional medicine based on both their own interests and the needs of the community. This helped them to address the disconnect between medical education and the sociocultural context they will work in. Our proposal was motivated by the idea that medical education should respond to the expectations and needs of patients and their communities, and that medical training should address the relationships between local culture and health outcomes.^35^

The medical students evaluated the structured introductory academic cycle in positive terms, mentioning that it gave them the confidence and motivation to approach the intercultural training. This suggests likely value in creating cultural safety training initiatives based on a structure that is familiar, logical, formal, easy-to-follow, and rigorous in the experience of medical students, allowing them more easily to internalise the content. It also highlights the need to refer to international guidelines and local legislation in cultural safety training.

Before the exposure to traditional medicine in this project, the medical students did not recognize the potential benefits of traditional practices. Medical students preferred the knowledge they received at university over the health practices they experienced at home. Students saw Western science as more advanced and civilized.^31^^,^^36^ Notwithstanding this, several students reported previous experience with traditional medicine, mostly through their families, as an enabling or motivating factor.

Importantly, the medical students near the end of their studies mentioned a complete lack of cultural safety training. According to Chitindingu,^37^ the increasing demand for traditional medicine in multicultural settings makes medical students show more interest in and enthusiasm for participating in intercultural training. This confirms the need to foster and document experiences in learning about cultural safety in Colombia.

### Limitations

A well-recognized limitation in medical education research is its risk of social desirability bias.^38^ Medical students could respond with the most socially desirable answers rather than their own point of view. We encouraged students to be sincere when writing down their narratives and assured them that their answers would not have any impact on any grade in any course. We recognize, nonetheless, the narratives are unlikely to reflect a full 360-degree account of the experience.

Our study is qualitative and involves only a small number of participants. The relative uniformity of narratives encourages us to think a larger size would not generate very different results, given the abovementioned likely bias. We report here only the student perspective (and not the community, schoolchild or teachers’ view), as the exercise set out to examine student motivation. The nature of the study means the results are likely to be influenced by our own values and beliefs, and our interpretation takes account of that limitation.

## Conclusions

Cultural safety should be a standard component in medical education in Colombia, which is currently quite distant from the cultural preferences of large segments of the population. Educators in similar settings may find our results informative in the design of training initiatives to improve professional interactions and to enhance respect for traditional medicine users.

We recommend anchoring cultural safety training in community voices, involving students and communities as co-authors of training content, providing formality and rigour to the content, and exploring prior student contact with traditional medicine. If time and resources are sufficient, the involvement of students in community-based programs offers a rich environment to encourage transformative learning.

Medical education about cultural safety is a necessary step in adapting health systems to the cultural expectations and preferences of the population. A transformative dynamic of these programs helps to generate attitudes of openness among medical students. We expect these students will be better prepared to recognize and address intercultural challenges in their future clinical practice.

### Acknowledgements

This study was financed by La Sabana University (Colombia), the CeiBA Foundation (Colombia) and the Fonds de recherche du Québec – Santé (Canada). The Cota Secretariat of Health sponsored part of the exercise through contract 640 of 2015. This did not influence the design and execution of the study.

Teachers Astrid Romero, Marcela Balcero, Mélida Valbuena, Patricia Hómes, and Sara Romero opened their classrooms to advance the activities of the project. One primary school (Institución Educativa Departamental Enrique Pardo Parra [Enrique Pardo Parra Departmental Educational Institution]) and two preschools (Sol Solecito [Sun Little-Sun] and Manitos Creativas [Creative Little-Hands]) allowed the implementation of the program. Camilo Correal, Rosa Durán, Sandra Espitia, Erwin Hernández, and Francisco Lamus contributed ideas to the project. Cassandra Laurie and Brenda Atkinson proofread the final version of the manuscript and supported its write-up.

### Conflict of Interest

The authors declare that they have no conflict of interests.

## References

[1] World Health Organization. Technical Report Series - The promotion and development of traditional medicine. Report of a WHO meeting (622). 1977. [Cited 29 March 2020]; Available from: http://apps.who.int/iris/bitstream/10665/40995/1/WHO_TRS_622.pdf. 102084

[2] World Health Organization. WHO traditional medicine strategy 2002-2005. 2002. [Cited 29 March 2020]; Available from: https://apps.who.int/medicinedocs/en/d/Js2297e/.

[3] World Health Organization. WHO Traditional medicine strategy: 2014-2023. 2013. [Cited 29 March 2020]; Available from: http://www.who.int/medicines/publications/traditional/trm_strategy14_23/en/.

[4] World Health Organization. Declaration of Alma-Ata international conference on primary health care, Alma-Ata, USSR, 6-12 September 1978. Development. 2004;47(2):159-61.

[5] Chan M (2008). Return to Alma-Ata.. Lancet.

[6] Bernal R, Cárdenas M. Race and ethnic inequality in health and health care in Colombia. In: Giuffrida A, editor. Racial and ethnic disparities in health in Latin America and the Caribbean. Washington, DC: Inter-American Development Bank; 2007.

[7] World Health Organization. Primary health care - Now more than even. 2008. [Cited 29 March 2020]; Available from: http://www.who.int/whr/2008/whr08_en.pdf.

[8] Williamson M, Harrison L (2010). Providing culturally appropriate care: a literature review.. Int J Nurs Stud.

[9] Allan B, Smylie J. First Peoples , second class treatment: The role of racism in the health and well-being of Indigenous peoples in Canada. 2015. [Cited 29 March 2020]; Available from: http://www.wellesleyinstitute.com/wp-content/uploads/2015/02/Summary-First-Peoples-Second-Class-Treatment-Final.pdf.

[10] Liaison Committee on Medical Education. Functions and structure of a medical school: standards for accreditation of medical education programs leading to the MD degree. 2017. [Cited 29 March 2020]; Available from: https://cacms-cafmc.ca/sites/default/files/documents/CACMS_Standards_and_Elements_-_AY_2019-2020.pdf.

[11] Vogel L (2018). Residency programs grapple with new Indigenous cultural safety training requirement.. CMAJ.

[12] Kirmayer LJ (2012). Rethinking cultural competence.. Transcult Psychiatry.

[13] Curtis E, Jones R, Tipene-Leach D, Walker C, Loring B, Paine SJ, Reid P (2019). Why cultural safety rather than cultural competency is required to achieve health equity: a literature review and recommended definition.. Int J Equity Health.

[14] Kurtz DLM, Janke R, Vinek J, Wells T, Hutchinson P, Froste A (2018). Health Sciences cultural safety education in Australia, Canada, New Zealand, and the United States: a literature review.. Int J Med Educ.

[15] Pimentel J, Zuluaga G, Isaza A, Molina A, Cockcroft A, Andersson N. Curriculum co-design for cultural safety training of medical students in colombia: protocol for a qualitative study. In: Costa AP, Reis LP, Moreira A, editors. Computer supported qualitative research. Cham: Springer; 2019.

[16] Mudur G (2001). Indian doctors decry proposal to teach traditional medicine.. BMJ.

[17] Lamus-Lemus F, Correal-Muñoz C, Hernandez-Rincon E, Serrano-Espinosa N, Jaimes-deTriviño C, Diaz-Quijano D, García-Manrique JG (2017). The pursuit of healthier communities through a community health medical education program.. Educ Health (Abingdon).

[18] Council for International Organizations of Medical Sciences. International ethical guidelines for biomedical research involving human subjects. Bull Med Ethics. 2002;(182):17-23. pmid: 14983848 14983848

[19] World Medical Association. World medical association declaration of Helsinki. JAMA. 2013;310(20):2191. 10.1001/jama.2013.281053 24141714

[20] Zuluaga G. Una ética para la investigación médica con comunidades indígenas. In: Vélez A, Ruiz A, Torres M, editors. Retos y dilemas de los comités de ética en investigación. Bogotá, Colombia: Editorial Universidad del Rosario; 2013.

[21] Alvarez M, Artiles L, Otero J, Cabrera N (2010). Priority setting in health research in Cuba, 2010.. MEDICC Rev.

[22] Hewitt-Taylor J (2012). Identifying, analysing and solving problems in practice.. Nurs Stand.

[23] Couillard J, Garon S, Riznic J. The logical framework approach-millennium. Project Management Journal. 2009;40(4):31-44.

[24] Braun V, Clarke V (2006). Using thematic analysis in psychology.. Qualitative Research in Psychology.

[25] Bozorgzad P, Negarandeh R, Raiesifar A, Poortaghi S (2016). Cultural safety: an evolutionary concept analysis.. Holist Nurs Pract.

[26] Parra L, Pacheco AM. Monologue or intercultural dialogue between medical systems? An educational challenge for medical sciences. Revista Ciencias de la Salud. 2006;4(2):110-121.

[27] Macaulay AC, Ing A, Salsberg J, McGregor A, Saad-Haddad C, Rice J, Montour L, Gray-Donald K (2007). Community-based participatory research: lessons from sharing results with the community: Kahnawake Schools Diabetes Prevention Project.. Prog Community Health Partnersh.

[28] Cargo M, Mercer SL (2008). The value and challenges of participatory research: strengthening its practice.. Annu Rev Public Health.

[29] Clark PG, Spaulding-Givens J. Can a low-complexity community-based project have transformative effects? Journal of Baccalaureate Social Work. 2016;21(1):127-49.

[30] Muñoz-Cano J, Maldonado-Salazar T, Bello J. Development projects for the training of intercultural competence by medical students. Revista de la Fundación Educación Médica. 2014;17(3):161–9.

[31] Arce-Antezana. The health professional training and the incorporation of Interculturality in the Curriculum Facultative. Gaceta Médica Boliviana. 2012;36(1):48–50.

[32] Bovill C. Students and staff co-creating curricula: an example of good practice in higher education? In: Dunne E, Owen D, editors. The student engagement handbook: practice in higher education. London, England: Emerald; 2014.

[33] World Federation for Medical Education. Basic medical education: WFME global standards for quality improvment. 2015. [Cited 29 March 2020]; Available from: https://wfme.org/standards/bme/.

[34] Bovill C, Cook‐Sather A, Felten P (2011). Students as co‐creators of teaching approaches, course design, and curricula: implications for academic developers.. International Journal for Academic Development.

[35] Prats-Gil E. Diversidad cultural y salud: una propuesta de formación inicial para profesionales de la educación. Educación Médica. 2004;8(1):18–41.

[36] Murillo J. Construction of intercultural competences in developing a learning experience proposal for first year medical students. Anales de la Facultad de Medicina. 2015;76:77–87.

[37] Chitindingu E, George G, Gow J (2014). A review of the integration of traditional, complementary and alternative medicine into the curriculum of South African medical schools.. BMC Med Educ.

[38] Gozu A, Beach MC, Price EG, Gary TL, Robinson K, Palacio A, Smarth C, Jenckes M, Feuerstein C, Bass EB, Powe NR, Cooper LA (2007). Self-administered instruments to measure cultural competence of health professionals: a systematic review.. Teach Learn Med.

